# Tophus resolution in patients with chronic refractory gout who have persistent urate-lowering responses to pegloticase

**DOI:** 10.1186/s13075-018-1782-x

**Published:** 2018-12-29

**Authors:** Brian F. Mandell, Anthony E. Yeo, Peter E. Lipsky

**Affiliations:** 1 Department of Rheumatologic and Immunologic Disease, Cleveland Clinic, Cleveland, OH USA; 2 Horizon Pharma, Lake Forest, IL USA; 3 AMPEL BioSolutions, LLC, 250 West Main Street, Charlottesville, VA 22902 USA

**Keywords:** Gout, Serum urate, Tophus, Pegloticase

## Abstract

**Background:**

Pegloticase is a recombinant mammalian uricase conjugated to polyethylene glycol approved in the United States for treatment of chronic refractory gout. It can profoundly decrease serum urate to < 1 mg/dl. In patients receiving pegloticase who did not generate high-titer antidrug antibodies (responders), the serum urate remained low for the duration of therapy, 6 months in the phase III clinical trials plus the open-label extension. The objective of this study was to assess the velocity of tophus resolution in subjects treated with pegloticase.

**Methods:**

Data from two randomized controlled trials of pegloticase in chronic refractory gout were analyzed. Tophi were assessed by computer-assisted measurements of standardized digital photographs. Subjects were designated as responders and nonresponders based on maintenance of serum urate < 6 mg/dl at months 3 and 6 of treatment. The projected time of complete resolution of all tophi was determined by linear regression analysis.

**Results:**

The mean total tophus area at baseline was 585.8 mm^2^ for responders, 661.5 mm^2^ for nonresponders, and 674.4 mm^2^ for placebo-treated patients. Complete resolution at 6 months of at least one tophus was achieved by 69.6% of 23 responders, 27.9% of 43 nonresponders, and 14.3% of 21 patients who received placebo. Complete resolution of all photographed tophi was achieved by 34.8% of biochemical responders, 11.6% of nonresponders, and 0% of placebo-treated patients. The mean velocity of resolution of all tophi was 60.1 mm^2^/month in responders with a mean projected time of complete resolution of 9.9 months (4.6–32.6 months). There was a significant inverse correlation between serum urate AUC and tophus resolution velocity (*r* = − 0.40, *P* = 0.0002), although considerable heterogeneity in the velocity of resolution was noted. The only patient characteristic that correlated with the velocity of tophus resolution was the baseline tophus area.

**Conclusions:**

Pegloticase treatment caused a rapid resolution of tophi in responders that correlated with the serum urate lowering associated with this therapy.

## Background

The tophus, a cardinal feature of gout, is a complex mass comprised of monosodium urate (MSU) crystals, a variety of immune and inflammatory cells, and a fibrous capsule [1, 2]. Tissue deposition of urate crystals initiates tophus formation with a local inflammatory and subsequent fibrotic tissue response. Clinically, it is difficult to dissect the various components of a tophus, and these may vary between different anatomical sites and different patients. Stimulation of immune/inflammatory system cells by MSU crystals may lead to chronic inflammation, pain, and tissue remodeling, including bone erosions [3, 4]. Much of the inflammatory capacity of urate crystals results from their capacity to stimulate the NLRP3 inflammasome with resultant production of IL-1β. The presence of tophi in patients with gout is associated with significantly decreased quality of life [5–12]. It has also been shown that clinically evident foot or ankle tophi are associated with muscle force deficits that may contribute to reduced muscular activation and consequent disuse muscle atrophy [13]. Tophaceous disease strongly predicts decreased hand function in patients with gout [14], because flexor or extensor tendon sheath deposition of urate can markedly reduce the ability to grasp and make a fist. The presence of tophi in patients with gout has also been associated with elevated mortality risk [15].

The most recent guidelines for treatment of gout from the American College of Rheumatology recognize the impact of tophi on patients and recommend that “the target serum urate should be lowered sufficiently to durably improve signs and symptoms of gout, including palpable and visible tophi detected by physical examination, and that this may involve therapeutic serum urate-lowering to below 5 mg/dl” [16].

Multiple studies have demonstrated the ability of urate-lowering therapy to reduce or resolve tophi [17–25]. However, results from most clinical trials indicate only modest decreases in tophi, perhaps owing to their relatively short duration [17, 21, 22]. Although there appears to be a significant correlation between the magnitude of serum urate reduction and resolution of tophi [26, 27], and although oral urate-lowering therapies are capable of decreasing serum urate from baseline by 50–60%, resulting in levels that are between 4 and 6 mg/dl [17, 20, 22], this may be insufficient for rapid tophus resolution.

Pegloticase is a recombinant mammalian uricase conjugated to polyethylene glycol approved in the United States for treatment of patients with chronic refractory gout. It profoundly decreases serum urate in biochemical responders to < 1 mg/dl [28]. The results of the pegloticase randomized clinical trials (RCTs) [18] provided the opportunity to determine the impact of persistent and markedly low levels of serum urate on the velocity of tophus resolution. Case reports and small-scale studies have indicated that treatment with pegloticase can produce rapid decreases in tophus mass in patients with gout [29–31]. Tophus reductions with pegloticase were also evaluated in the RCTs of this agent [19], but this analysis combined results from patients who responded to this treatment with sustained reductions in serum urate and those who did not. The present analysis focuses on tophus reduction in patients who responded with persistent urate lowering to pegloticase and compares results from this group with those from patients who did not have a persistent response (nonresponders) and those who received placebo.

## Methods

### Design of pegloticase clinical trials

Results from two identical RCTs of pegloticase were analyzed (NCT00325195, NCT01356498) [18, 28]. The methods for these studies have been described in detail elsewhere [18, 28] and are summarized only briefly herein. These studies received institutional review board approval at each site. Written informed consent and Health Insurance Portability and Accountability Act assurances were completed for each participant before enrollment. The design and conduct of the study complied with the Declaration of Helsinki.

### Patients

Patients were ≥ 18 years of age with chronic refractory gout, defined as baseline serum urate ≥ 0.476 mmol/L (8 mg/dl), and at least one of the following: (1) at least three self-reported gout flares during the previous 18 months, (2) at least one tophus, and/or (3) gouty arthropathy, defined clinically or radiographically as joint damage caused by gout, and (4) contraindication to treatment with allopurinol or history of failure to normalize urate despite ≥ 3 months of treatment with the maximum medically appropriate allopurinol dose. Patients were randomized to 6 months of treatment with intravenous infusions of either pegloticase 8 mg at each infusion (8 mg q2w), pegloticase 8 mg alternating with placebo (q4w), or placebo [28].

### Tophus assessment

Tophus assessment was carried out using Computer-Assisted Photographic Evaluation in Rheumatology methodology [32]. Each study site was supplied with a calibrated digital camera, digital media storage cards, preprinted templates for hand and foot photography, and a light stand. A designated individual was trained and took all photographs at each site.

Photographs of the hands and feet were taken for each patient at baseline and repeated at weeks 13, 19, and 25 of the RCTs using the same views of the tophus sites identified at baseline. Serial photographs of up to two additional regions were taken at the discretion of the investigator, based on other tophi identified at baseline. Digital media cards containing the photographs were sent to RadPharm (Princeton, NJ, USA), where central readers, who were blinded to treatment assignment, evaluated the photographs prospectively and identified sites of tophi present at the start of treatment as well as response to therapy.

### Endpoints

#### Biochemical responder analysis

A responder (primary endpoint for the RCTs) was defined as a patient with plasma UA < 0.36 mmol/L (6 mg/dl) for ≥ 80% of the 15 measurements taken during periods of intensive monitoring from the week 9 infusion to just before the week 13 infusion (bracketing 3 months after the initiation of infusions) and from the week 21 infusion to the week 25 final study visit (bracketing 6 months after the initiation of infusions).

### Tophus resolution

Two measures were used to assess the effects of pegloticase treatment on the photographed tophi. The first was the complete resolution of at least one tophus without development of new tophi or progressive enlargement of any other tophus. This was the definition employed in an earlier evaluation of the effects of pegloticase on tophi [19]. The second measure was based on the total number of photographically identified tophi at baseline and assessed as total area of all tophi combined. In this analysis, a response was defined as complete resolution of all of these tophi.

### Serum urate exposure

AUCs of serum urate during the 6-month study were calculated according to the equation:

$$ AUC={\int}_a^b\mathrm{F}\left(\mathrm{X}\ \right)\mathrm{dX}, $$

where *a* and *b* are points on the *x*-axis and F(X) is the integral of function using SAS software (SAS Institute, Cary, NC, USA). Because the curves were not always continuous functions, the trapezoidal rule was employed to approximate the definitive integral.

### Analysis populations

Results from three groups were evaluated: (1) patients treated with pegloticase q2w who were responders (defined above), (2) patients who received pegloticase the entire 6 months but did not meet the criteria for a response (nonresponders), and (3) patients who received placebo.

Results for responders were also subdivided on the basis of baseline tophus burden into tertiles as low (total baseline tophus area < 668 mm^2^), medium (baseline tophus burden 688–1690 mm^2^), and high (baseline tophus burden > 1690 mm^2^), and the velocity of tophus resolution was determined for each of these groups.

### Data analysis

Kaplan-Meier analysis was used for evaluation of time to events. Because the number of subjects in each group was small, nonparametric tests were used. Unpaired two-sample Wilcoxon tests were used when comparing groups. One-way analyses of variance were employed to derive group means for all variables. Between-group differences for categorical variables were determined by Fisher’s exact tests. The projected time of complete resolution of all tophi was determined by linear regression using SAS software. The relationship between serum urate exposure and velocity of tophus reduction was evaluated with a Pearson product-moment correlation, and significance was determined with a *t* test. *P* < 0.05 was the accepted level of significance for all significance tests.

## Results

### Patients

The analysis included results from 66 patients who received pegloticase every 2 weeks (q2w) (23 responders, 43 nonresponders, and 21 patients who received placebo). The baseline demographic and clinical characteristics of these patients are summarized in Table 1. Except for age, there were no significant differences among the three groups for any demographic or clinical variable.

**Table 1:** Baseline demographic characteristics of the three groups of patients included in the analysis

Characteristic	Responders (*n* = 23)	Nonresponders (*n* = 43)	Placebo (*n* = 21)	*P* value
Age, years, mean (SD)	63.0 (14.1)	53.5 (14.0)	56.8 (10.5)	0.043
Sex, n	17 M, 6F	37 M, 6F	17 M, 4F	0.48
Disease duration, years, mean (SD)	17.0 (17.2)	15.1 (9.8)	14.9 (9.9)	0.97
sUA, mg/dl, mean (SD)	10.22 (1.76)	10.50 (1.62)	10.19 (1.82)	0.68
Number of tophi assessed	93	98	68	NC
Total tophus area, mm^2^, mean (SD)	585.8 (795.0)	661.5 (1298.7)	674.4 (1011.6)	0.32
Comorbidities, n				
Hypertension	16	30	17	0.60
Dyslipidemia	14	17	10	0.25
Diabetes mellitus	4	7	4	0.96
Coronary artery disease	4	3	2	0.41

*Abbreviations: M* Male, *F* Female, *NC* Not computed

*P* value is for comparison of responders, nonresponders, and placebo

### Tophus resolution

**Fig. 1:**
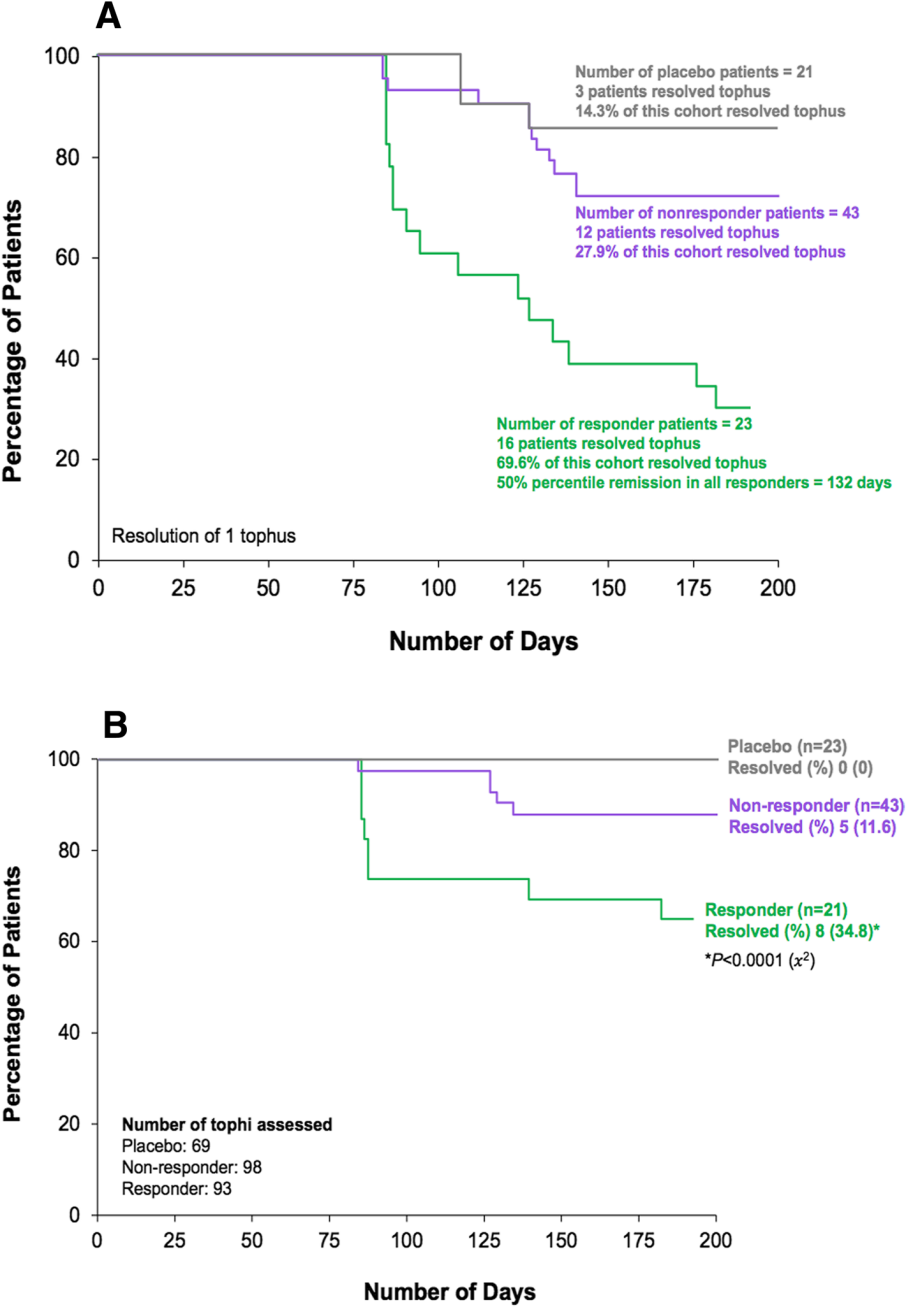
**a** Kaplan-Meier analysis of time to complete resolution of at least one tophus without development of new tophi or progressive enlargement of any other tophus. **b** Kaplan-Meier analysis of time to complete resolution of all tophi

The mean total tophus area (mean [SD]) at baseline was 585.8 mm^2^ (795.0) for responders, 661.5 mm^2^ (1298.7) for nonresponders, and 674.4 mm^2^ (1011.6) for placebo patients (*p* = 0.32). Complete resolution of at least one tophus without development of new tophi or progressive enlargement of any other tophus was achieved by 69.6% of responders, and the median time to resolution for this subgroup of patients was 132 days. This goal was achieved by 27.9% of nonresponders with a median time to resolution of 162 days; 14.3% of patients who received placebo achieved this outcome, and the median time to resolution was 171 days (Fig. 1a). The percentage of responders who achieved this criterion was significantly higher than for nonresponders (*P* = 0.001) or patients treated with placebo (*P* = 0.0006). The response rate for nonresponders was not significantly different from that for patients who received placebo (*P* = 0.24).

Complete resolution of all tophi photographed was achieved by 34.8% of responders (93 tophi assessed) in the 6 months of the RCT, 11.6% of nonresponders (98 tophi assessed), and 0% of placebo-treated patients (69 tophi assessed) (Fig. 1b). The percentage of responders who achieved this criterion was significantly higher than for nonresponders and patients treated with placebo (*P* < 0.001). The frequency of subjects who completely resolved tophi for nonresponders was not significantly different from that for patients who received placebo (*P* = 0.16).

### Velocity of tophus resolution

**Fig. 2:**
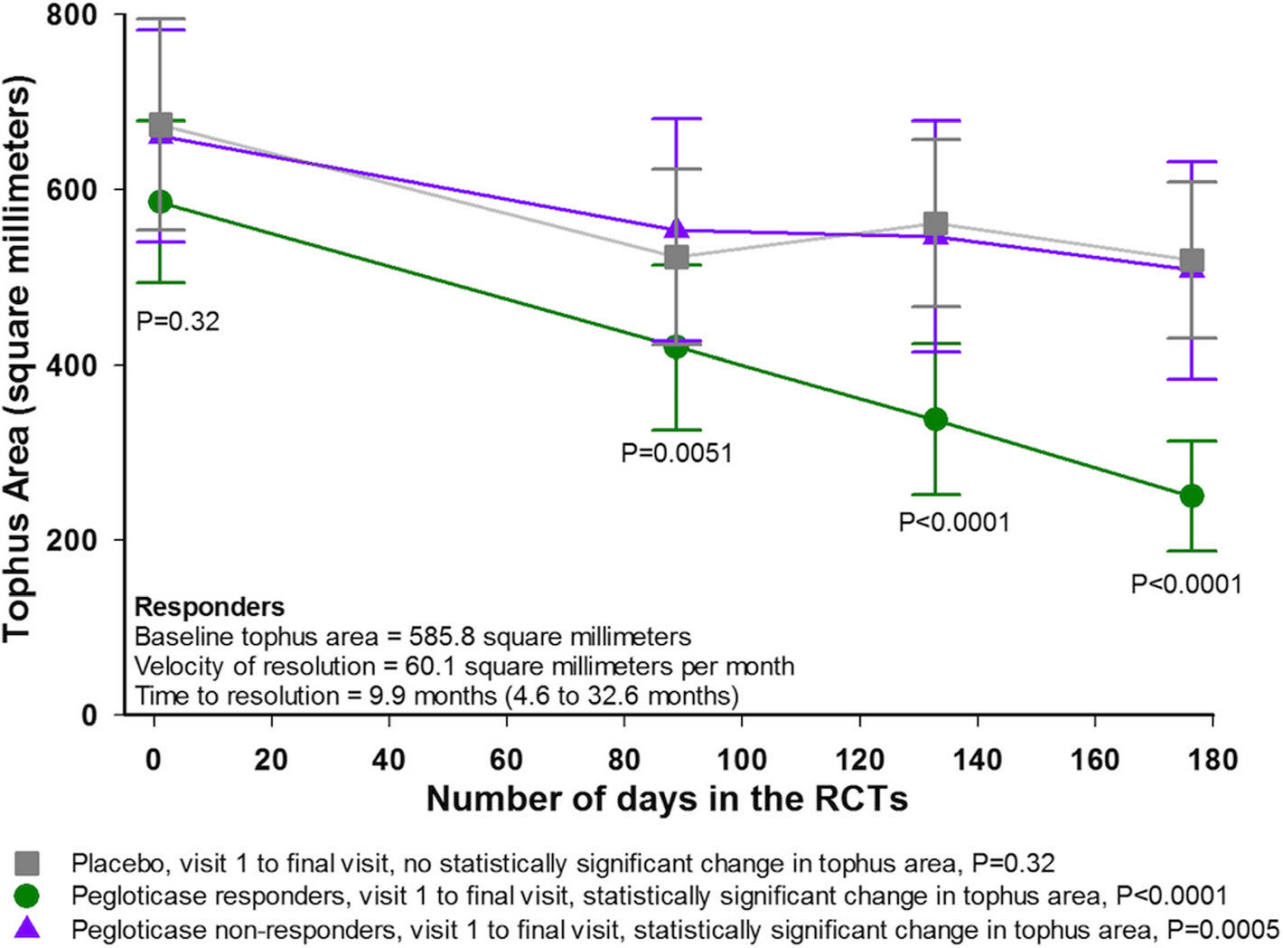
Mean (±SD) tophus area over time for the three groups of patients evaluated

Comparison of results for responders, nonresponders, and patients who received placebo indicated significant differences among groups for tophus area at all postbaseline assessments (Fig. 2) (all *P* ≤ 0.01). Reductions from baseline to the end of 6 months were significant for responders and nonresponders (*P* < 0.0001 and *P* = 0.0005, respectively), but not for those who received placebo (*P* = 0.32). The velocity of tophus resolution for the responders was 60.1 mm^2^ per month, with a projected time of full resolution of 9.9 months. The velocity of tophus resolution was statistically different from that for the nonresponders (22.3 mm^2^ per month, *P* = 0.053) and for patients treated with placebo (28.4 mm^2^ per month, *P* = 0.0034). The projected time to complete tophus resolution in biochemical responders was 9.9 months.

### Exposure to different serum urate treatment groups

**Fig. 3:**
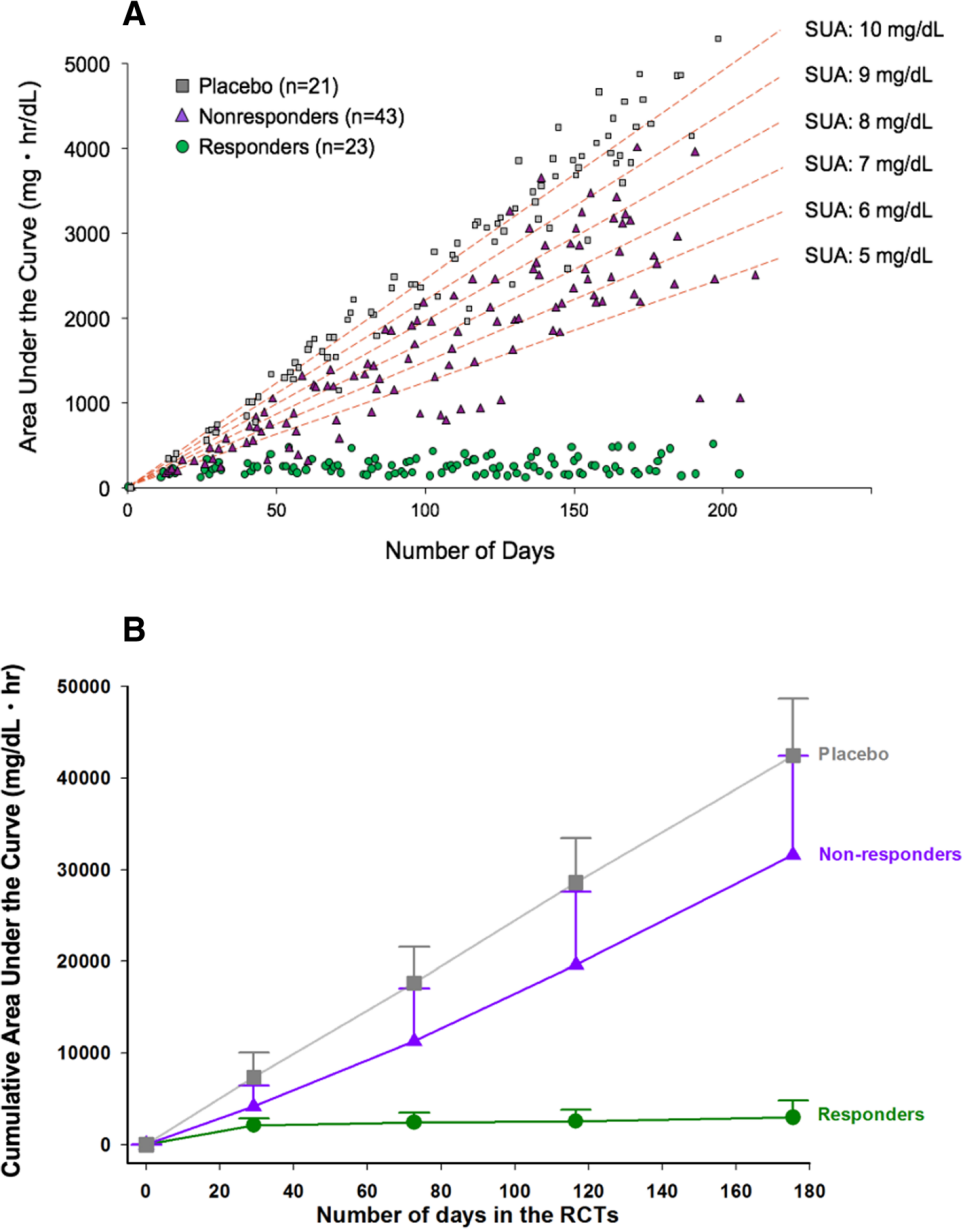
**a** Cumulative serum urate AUC values for individual patients at different time points and projected cumulative serum urate AUCs for different mean serum urate values over the course of the study. **b** Mean AUC [±SD] for serum urate in the three groups included in the analysis

The mean (SD) serum urate values at the end of treatment were 0.49 mg/dl (01.41) (0.029 mmol/L) for the biochemical responders, 8.47 mg/dl (3.61) (0.504 mmol/L) for the nonresponders, and 10.25 mg/dl (1.23) (0.610 mmol/L) for the patients who received placebo (*P* < 0.0001 for responders vs the other two groups). The most unbiased approach for determining serum urate exposure over the course of the RCTs was determination of the AUC for all of the measurements made during the study (Fig. 3a). The mean (SD) value for this measure for biochemical responders was 2907.0 mg/dl·h (1834.3), and it was significantly lower than values for either nonresponders (31,615.6 [10,814.9]) (*P* < 0.0001) or patients treated with placebo (42,442.6 [6234.1]) (*P* < 0.0001) (Fig. 3b).

### Relationship between serum urate exposure and velocity of tophus reduction

**Fig. 4:**
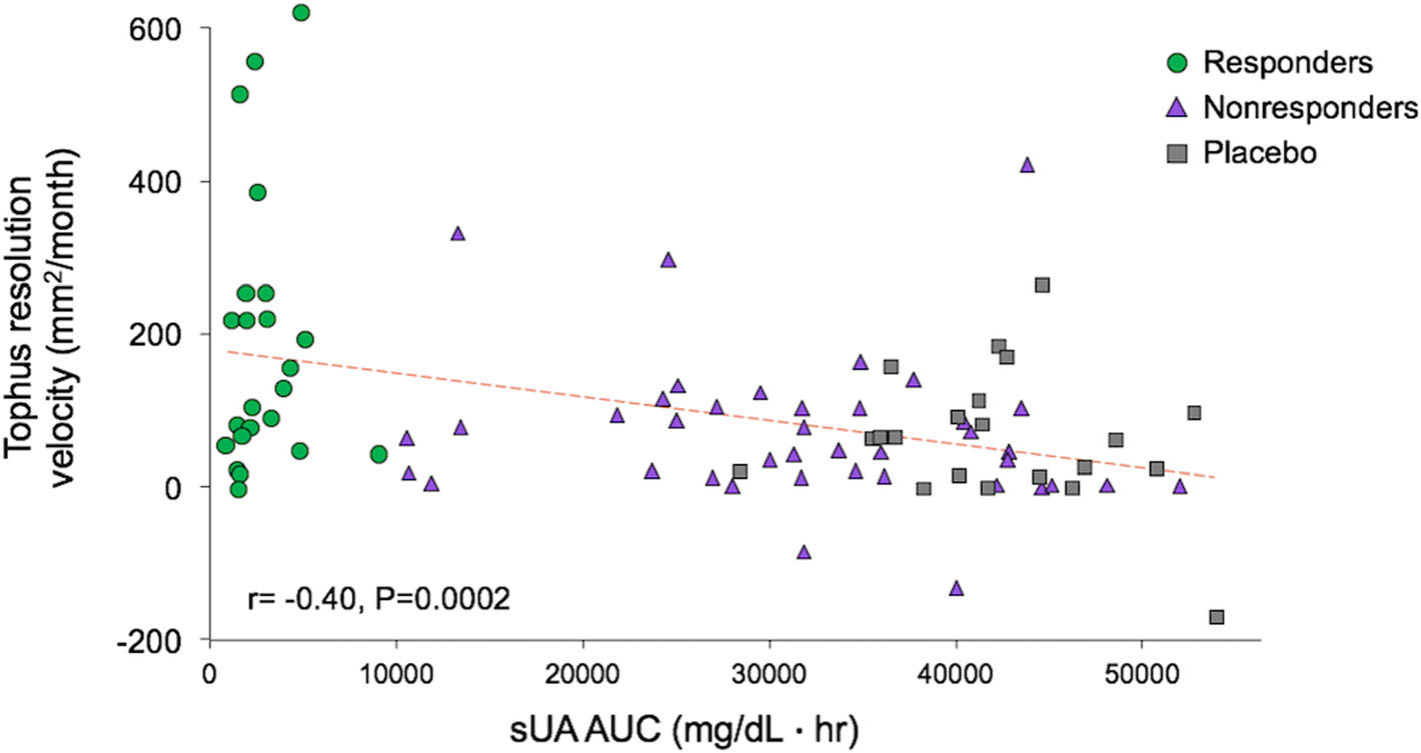
Serum urate AUC vs velocity of tophus reduction in responders, nonresponders, and placebo-treated patients

Combined analysis of results from all three groups of patients included in this analysis indicated a significant inverse correlation between serum urate AUC and tophus resolution velocity (*r* = − 0.40, *P* = 0.0002) (Fig. 4). The mean time to resolution of all tophi for biochemical responders was 9.9 months (range, 4.6 to 32.6 months). Clinical features, including age, body mass index, gender, race, and tophus location, did not significantly influence velocity of tophus resolution.

### Baseline tophus burden and tophus reduction

**Fig. 5:**
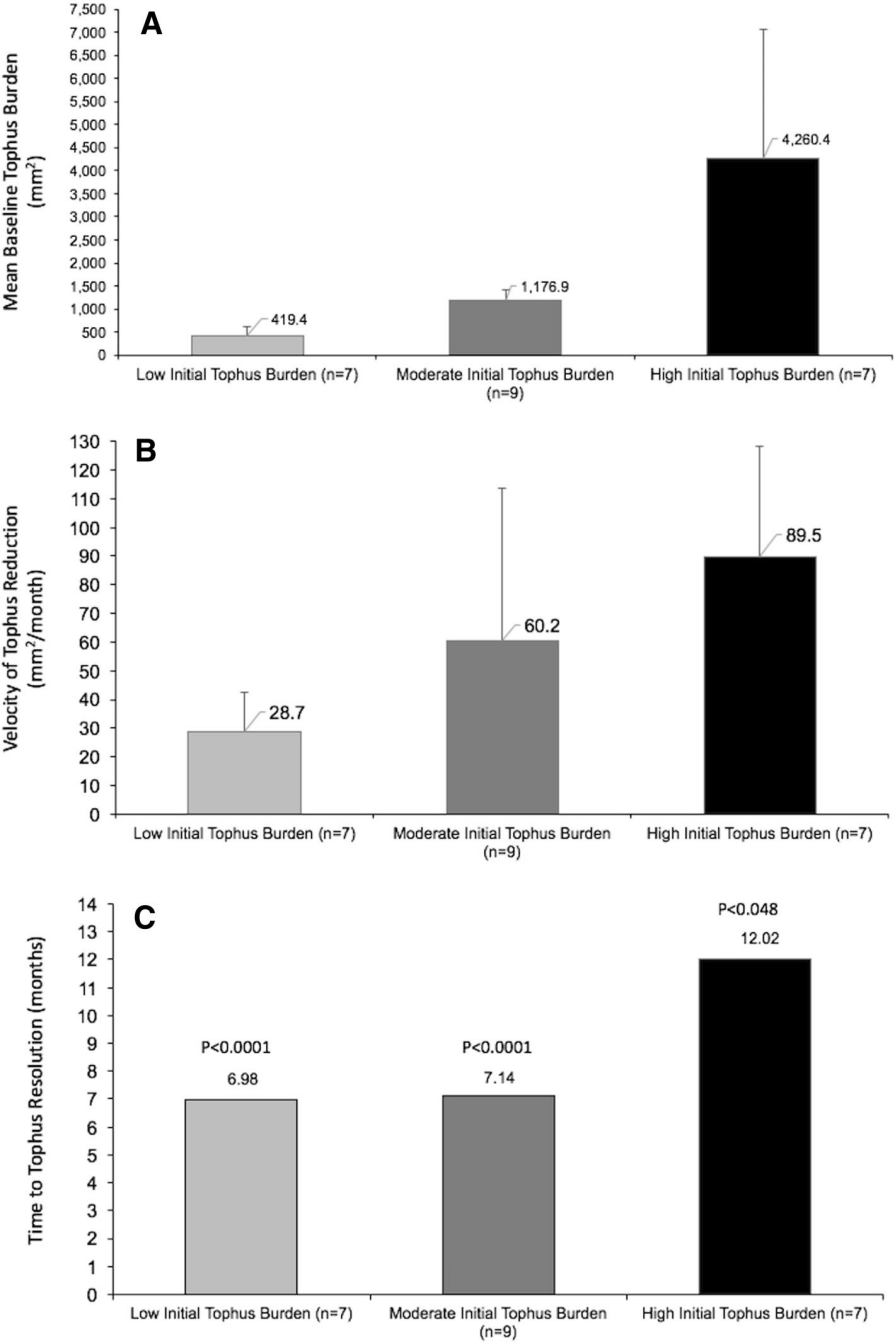
**a** Baseline tophus areas for biochemical responders with low, medium, and high tophus burden at baseline. **b** Velocity of tophus resolution for patients the three groups. **c** Mean time to tophus resolution for patients with low, medium, and high tophus burden at baseline. Error bars are SDs

In responders, the mean (SD) baseline tophus areas at baseline were 419.4 mm^2^ (202.4) for patients with low baseline tophus burden, 1176.9 mm^2^ (238.7) for those with moderate tophus burden, and 4260.4 mm^2^ (2784.9) for those with high baseline tophus burden (Fig. 5a). The mean (SD) velocity of tophus resolution was 28.7 (13.6) mm^2^/month, and the mean time to tophus resolution was 6.98 months (range, 5.02 to 10.0 months) for patients with low baseline tophus burden (*P* < 0.0001 compared with those with high tophus burden). For those with moderate baseline tophus burden, these values were 60.2 (53.5) mm^2^/month and 7.14 months (range, 5.21 to 10.1 months) (*P* < 0.0001 compared with those with high tophus burden), and for those with high baseline tophus burden, they were 89.5 (38.7) mm^2^/month and 12.02 months (range, 6.73 to 27.91 months) (*P* = 0.0477) (Fig. 5b and c).

## Discussion

This analysis demonstrates that about one-third of patients who responded biochemically to pegloticase as defined in the RCTs achieved resolution of all tophi within the 6 months of the RCTs. This goal was also achieved by 11.6% of pegloticase-treated patients who did not have sustained reductions in sUA to < 6 mg/dl for the duration of the study, but in none of those receiving placebo. Correlational analysis showed further that reduction in exposure to serum urate achieved with pegloticase was correlated with the velocity of tophus resolution.

Previous studies have shown that treatments achieving more modest lowering of the serum urate can reduce tophus burden in patients with gout, but it is difficult to directly compare results from those clinical trials with those from the present analysis. In other studies, results are generally reported as the percentage of patients who achieved resolution of a single target tophus [17, 20] or reduction of all tophi [22]. However, neither the tophus burden at baseline nor the velocity of tophus resolution was reported in these trials. One exception to this generalization is a trial in which 63 patients with microscopically confirmed tophaceous gout were treated with allopurinol, benzbromarone, or the combination of these two agents [23]. Results from that study, like those reported here, indicated a significant inverse relationship between the serum urate level during therapy and the velocity of tophus reduction. The mean time from onset of urate-lowering therapy to the disappearance of the target tophus for all patients was 20.8 months (range, 6–64 months). This is much longer than that reported in our analysis, and it is almost certainly related to the fact that the average on-treatment serum urate for the patients evaluated ranged from 3.97 to 5.37 mg/dl in different treatment arms of the trial reported by Perez-Ruiz vs an end-of-treatment value of 0.49 mg/dl for the biochemical responders in the present analysis. The highest velocity of tophus reduction (decrease in diameter) was 1.53 mm per month, and the initial tophus diameter for patients in the Perez-Ruiz study was 14.5 mm. Assuming that the tophi were approximately circular, this equates to a reduction in area of 33.2 mm^2^ per month, which is approximately one-half of that for the responders in this analysis and only slightly higher than that for the nonresponders.

Nonresponders to pegloticase typically have an initial profound reduction in serum urate that is followed by a gradual increase in serum urate over approximately 6 weeks. This loss of response is strongly associated with development of high titer of antibodies to pegloticase [28, 33]. The results of the present analysis suggest that these patients may have received some benefit from pegloticase with respect to tophus reduction, because complete resolution of at least one tophus without development of new tophi or progressive enlargement of any other tophus was achieved by 27.9% of this group, and resolution of all photographed tophi was achieved by 11.6%. This is consistent with previous observations in patients treated with pegloticase and the suggestion that even transient lowering of serum urate levels may decrease tophus volume because they are dynamic structures and begin to decline in size as soon as serum urate levels are decreased [30].

A final point worth noting is that the incomplete tophus resolution in any study employing photographs for this determination may underestimate the benefit of serum urate-lowering treatment. A typical tophus consists of a mostly acellular crystalline core surrounded by a corona zone with multiple cell types, including macrophages, mast cells, and lymphocytes, and an outer, loose fibrovascular zone [3, 34]. The reductions in tophus size reported here probably reflect decreases in the mass of MSU crystals, which might be complete but still leave chronic inflammation and a fibrotic “shell,” which may take various periods of time to resolve. The heterogeneous composition of tophi along with interindividual variations in urate solubilization likely contributed to the high interpatient variability in velocity of tophus resolution. Subject characteristics, including age, body mass index, sex, race, and tophus location were not correlated with velocity of tophus resolution. However, there was a significant positive correlation between initial tophus area and velocity of resolution. Presumably, larger tophi may have a greater relative component of urate crystals that can be mobilized rapidly by pegloticase treatment.

The limitations of this study include the post hoc nature of the analysis, the assessment of only subcutaneous tophi, and the use of photographic rather than clinical measures of tophus area. It is possible that deeper tophi respond with a different velocity than subcutaneous ones and that clinical evaluation of tophi might have been more effective by allowing assessment of the consistency of the lesion as well as its dimensions. Despite these caveats, the data contribute to understanding of the relationship between serum urate burden and tophus resolution and also indicate the heterogeneity in tophus resolution.

## Conclusions

Pegloticase treatment causes a rapid resolution of tophi in responders, as predicted from the profound and persistent serum urate lowering associated with this therapy. Even transient reductions in serum urate observed in nonresponders may result in complete resolution of tophi in some patients over the course of 6 months or less.

## Abbreviations

MSU: Monosodium urate crystals
q2w: Every 2 weeks
q4w: Every 4 weeks
RCT: Randomized clinical trial
sUA: Serum uric acid
UA: Uric acid
